# Understanding student optimism through academic grit, social connectedness, and resilience

**DOI:** 10.3389/fpsyg.2026.1595580

**Published:** 2026-05-19

**Authors:** Yinghui Sun, Li Wang

**Affiliations:** School of Nursing, Qilu Medical University, Zibo, China

**Keywords:** academic grit, mixed methods, optimism, resilience, social connectedness, structural equation modeling, thematic analysis, university students

## Abstract

This study investigated the interrelationships between academic grit, social connectedness, resilience, and optimism in university students using an explanatory sequential mixed-methods design. Quantitative structural equation modeling (SEM) of survey data (*N* = 372) revealed that academic grit and social connectedness were significantly associated with resilience, which, in turn, statistically accounted for their associations with optimism. Qualitative thematic analysis of student diaries (*n* = 40) provided rich contextual insights, illustrating how students navigate academic challenges, utilize social support, and develop resilience as a self-reflective process linked to optimism. These findings highlight the intervening role of resilience in connecting the benefits of perseverance and social bonds to a positive outlook during academic life. While the cross-sectional nature of the data precludes causal claims, the findings align with a theoretical framework where resilience, grit, and social connectedness interact within university settings to enhance student well-being and academic success. Educational implications include interventions targeting resilience-building, social support networks, and the cultivation of grit to promote student optimism and overall positive academic trajectories.

## Introduction

Higher education is a demanding yet critical phase of development. University students encounter unique academic, social, and personal challenges, from intense coursework and examinations to new social environments and the establishment of independence (Aburn et al., 2016; Lam and Zhou, 2019; Luthar et al., 2000). Understanding the psychological resources that enable students to thrive, rather than merely survive, is therefore essential. Academic grit, social connectedness, resilience, and optimism are increasingly recognized as key factors associated with positive academic and well-being outcomes (Alhadabi and Karpinski, 2020; Anderson et al., 2018).

Academic grit, defined by perseverance and commitment to long-term goals (Duckworth et al., 2007), represents the proactive tenacity needed to navigate the prolonged challenges of academic life. Critically, grit is a “marathon” construct; it describes the duration of effort toward a specific vision, even in the absence of immediate failure. Gritty students demonstrate sustained dedication to their studies, allowing them to persist through setbacks and maintain academic focus (Datu, 2021; Wolters and Hussain, 2015). Social connectedness, reflecting the quality of interpersonal relationships, offers crucial support and belonging for students adapting to university. Strong social networks provide emotional and practical resources, reducing the isolation of academic stress (Allen et al., 2008; Çiçek, 2021).

Resilience, the capacity for positive adaptation to adversity (Luthar et al., 2000; Masten and Cicchetti, 2016), is a reactive psychological mechanism. Unlike grit, which is goal-specific and steady, resilience is process-specific and triggered by shocks (Aburn et al., 2016; Bhamra et al., 2011); it is the “elasticity” that allows a student to recover their baseline functioning after an acute setback, such as a failed assessment or personal crisis (Smith et al., 2008). Resilient students effectively recover from difficulties, maintain emotional balance, and continue pursuing their goals despite obstacles (Smith et al., 2008). Optimism, the generalized expectation of positive future outcomes (Scheier and Carver, 1985), underpins these adaptive capacities. An optimistic outlook promotes proactive coping, enhances motivation, and buffers against stress (Carver and Scheier, 2023; Rand et al., 2020; Tetzner and Becker, 2018).

Despite the established importance of these individual constructs, a critical gap remains in understanding the underlying pathways that link them. Most existing research treats grit or social support as isolated predictors, failing to account for the internal mechanisms—specifically the mediating function of resilience—that relate these resources to a stable optimistic outlook. Although the current study employs a cross-sectional design that precludes definitive causal claims, the hypothesized directional model is grounded in established frameworks. Specifically, the Conservation of Resources (COR) theory (Hobfoll, 1989) suggests that personal and social assets (grit and connectedness) serve as foundational resources that precede functional capacities like resilience. Furthermore, the Broaden-and-Build theory (Fredrickson, 2001) posits that the ability to recover from stress (resilience) expands one’s cognitive repertoire, which is theoretically linked to a more optimistic future orientation.

Identifying these specific psychological “bridges” is essential for moving beyond general observations toward targeted interventions. Without a clear map of how grit and social bonds associate with optimism, educational support programs remain speculative rather than evidence-based. The feasibility of this research is supported by a robust explanatory sequential mixed-methods design. The university environment provides an ideal “natural laboratory” for this study, as students are frequently exposed to stressors that require the activation of both internal traits and external resources. By examining these statistical associations through Structural Equation Modeling (SEM) and supplementing them with qualitative diaries, this study seeks to clarify the complex interplay between perseverance, community, and outlook. This mixed-methods approach ensures that the relationships are measured with both statistical precision and contextual accuracy, moving beyond general observations toward a more nuanced understanding of student thriving.

## Literature **review**

### Academic grit and its influence on student achievement

Academic grit, defined by perseverance and passion for long-term goals, is a critical psychological construct in education (Duckworth et al., 2007). Unlike innate talent, grit emphasizes sustained dedication and effort in overcoming obstacles (Datu, 2021; Tang et al., 2019). This concept has garnered significant interest due to its strong links with academic achievement, motivation, and persistence across diverse learning environments (Duckworth et al., 2007; Lam and Zhou, 2019). Beyond performance, grit intertwines with motivational and psychological factors that shape broader educational outcomes (Credé, 2018; Fathi and Behzadpoor, 2025; Whipple and Dimitrova-Grajzl, 2021). Recent person-centered research has further clarified this construct by identifying distinct profiles—such as those characterized by both high effort and interest—that represent an optimal configuration of personal resources. From the perspective of Conservation of Resources (COR) theory, these high-grit profiles enable students to manage academic demands and emotional stressors more effectively (Liu et al., 2025).

Grit comprises perseverance of effort and consistency of interest (Duckworth et al., 2007). Perseverance is the ability to maintain effort despite setbacks, while consistency of interest is sustained commitment to long-term aspirations. This aligns with goal-setting theory, which posits that specific, challenging goals enhance success by fostering sustained effort and focus (Christopoulou et al., 2018; Locke and Latham, 1990). Essentially, grit prioritizes committed hard work over inherent aptitude in achieving goals (Duckworth and Quinn, 2009; Lam and Zhou, 2022). Theoretically, grit influences optimism through the mechanism of self-efficacy; as students successfully persist through long-term challenges, they develop a sense of mastery that leads to a generalized expectation of future success. This suggests that grit is not just a predictor of performance, but a cognitive foundation for a positive outlook (Alhadabi and Karpinski, 2020).

Empirical evidence strongly supports the positive relationship between grit and academic performance across various educational levels and disciplines (Fathi et al., 2024; Fernández-Martín et al., 2020; Lam and Zhou, 2022; Mohammad Hosseini et al., 2024; Pawlak et al., 2025). Studies indicate that grittier students utilize more effective self-regulated learning strategies, which enhance academic outcomes in higher education (Wolters and Hussain, 2015). This positive correlation is consistent across settings; higher grit scores correlate with greater academic engagement and superior results (Hodge et al., 2018). Systematic reviews further confirm grit’s role as a robust predictor of success throughout K-12 and university contexts (Lam and Zhou, 2019). Notably, grit predicts achievement even when controlling for cognitive abilities, particularly in demanding fields like science, suggesting it serves as a protective factor against academic challenges (Alhadabi and Karpinski, 2020; Bazelais et al., 2016).

Beyond performance, grit significantly relates to motivational constructs like self-efficacy and intrinsic motivation (De La Cruz et al., 2021; Usher et al., 2019). While self-control and conscientiousness are relevant, grit uniquely sustains long-term motivation, especially without immediate rewards, crucial for challenging academic tasks (Werner et al., 2019). Grittier students are more likely to set and pursue long-term academic goals, reinforcing goal-setting theory in fostering persistence and motivation (Locke and Latham, 1990; Muenks et al., 2018). Furthermore, the link between grit and optimism can be explained by the fact that grittier individuals are less likely to perceive setbacks as permanent, a hallmark of an optimistic explanatory style (Carver and Scheier, 2014). By maintaining effort, students validate their ability to influence outcomes, which directly sustains their optimism.

The interplay between grit and resilience is fundamental to overcoming academic adversity. Although these are theoretically adjacent constructs with significant conceptual overlap, their precise directional relationship remains a subject of ongoing debate (Clark and Malecki, 2019; Datu, 2021). This study maintains a clear conceptual boundary by distinguishing their functional roles: academic grit is viewed as a proactive, long-term commitment to specific goals (“stamina”), whereas resilience is a reactive capacity for positive adaptation and emotional recovery (“elasticity”) following adversity. Grit drives the sustained effort toward degree completion (the “marathon”), while resilience manages acute psychological shocks, such as failing an assignment (the “bounce-back”). Research suggests that domain-specific grit is significantly associated with school satisfaction, providing the psychological buffer necessary for resilience (Clark and Malecki, 2019). Rather than serving as a synonym, grit provides the motivational “why” that necessitates resilient recovery (Duckworth et al., 2007; Usher et al., 2019); a gritty student is more likely to activate recovery processes because their long-term passion renders the goal worth the effort of recuperation (Datu, 2021; Hodge et al., 2018). Grittier students typically view challenges as growth opportunities (Lee, 2017), and longitudinal evidence indicates that growth mindsets foster grit development, which in turn strengthens resilience (Tang et al., 2019). Specifically, grit functions as a critical mediating mechanism in self-regulation, bridging the gap between a growth mindset and the capacity for academic delay of gratification (Zhao et al., 2023). By sustaining a consistent trajectory, grit provides the behavioral framework within which resilience functions as a corrective mechanism.

In rigorous academic environments, grit is essential for navigating stressors such as examinations and complex assignments (Chang, 2014). Grit predicts achievement independently of conscientiousness and engagement, highlighting its unique utility in settings where persistence is paramount (Steinmayr et al., 2018). Targeted interventions, such as mindfulness-based cognitive therapy, can enhance grit by fostering the perseverance required to manage academic demands (Rusadi et al., 2023). Furthermore, promoting optimism may indirectly strengthen both grit and resilience, suggesting the efficacy of multifaceted cultivation strategies (Pyo et al., 2024). Ultimately, academic grit is a foundational driver of student achievement, providing the sustained motivation and engagement necessary to thrive amidst adversity.

### Social connectedness and its impact on academic success and well-being

Social connectedness, defined as the sense of belonging and emotional proximity within social networks, is fundamental to psychological adaptation (Lee and Robbins, 1995). Rooted in self-psychology, it represents the perceived capacity to establish and maintain meaningful relationships, which is vital for navigating life’s challenges (Kohut, 1984). Theoretically, Social Support Theory explains how this “safety net” reduces psychological burdens; by knowing a reliable network is accessible, students maintain optimism, feeling equipped to handle stressors through collective resources rather than individual effort alone (Allen et al., 2008; Bailey et al., 2018; Van Bel et al., 2009).

In academic settings, social connectedness mitigates stress and facilitates the management of rigorous demands (Bond et al., 2007; Tang et al., 2025). Peer relationships are especially salient, as shared experiences reduce isolation and provide opportunities for collaborative coping strategies (Apker, 2022; Grapin et al., 2016; Oddone Paolucci et al., 2021; Pym et al., 2011). Furthermore, teacher support serves as a critical resource that acts as a precursor to academic buoyancy, directly reducing burnout and negative emotions (Derakhshan and Fathi, 2025; Li et al., 2025). When students perceive a supportive instructional environment, they more effectively leverage social bonds to navigate setbacks, thereby reinforcing positive future expectations.

Strong social networks also ease the transition into higher education by countering the isolation associated with heightened academic rigor (Mishra, 2020; Pym et al., 2011; Vasileiou et al., 2019). Beyond the campus, family support remains a primary determinant of achievement. Students who perceive support from both peers and family demonstrate superior academic engagement and focus (Bradley et al., 2021; Moreira et al., 2018; Roksa and Kinsley, 2019). This dual support system offers the emotional reassurance necessary to bolster resilience and ensure long-term academic success (Gray et al., 2013; Guo et al., 2025; Jose et al., 2012; Shengyao et al., 2024; Turki et al., 2018).

A primary psychological benefit of connectedness is enhanced resilience, or the capacity to recover from adversity. Students deeply integrated into their social and academic communities are better equipped to navigate stressors and maintain persistence (Allen et al., 2008; Çiçek, 2021; Nitschke et al., 2021). These resources are foundational for rebounding from difficulties and sustaining academic momentum (Abubakar and Dimitrova, 2016; Meng et al., 2018; Versteeg et al., 2022). Furthermore, validation from a supportive community fosters self-worth, which students internalize as optimistic beliefs regarding their future capabilities (Olasupo et al., 2021).

Social bonds also significantly contribute to life satisfaction and motivation (Jose et al., 2012; Olasupo et al., 2021; Rehman et al., 2023). Connected students tend to frame academic challenges as growth opportunities rather than threats (Ergün-Başak and Can, 2018). This often involves “adaptive social navigation,” where students strategically manage their networks—drawing on peers for practical assistance and family for emotional stability—to meet specific needs (Bradley et al., 2021). This active engagement effectively buffers academic stress, positioning social connectedness as a critical predictor of both resilience and optimism.

### Resilience as a key psychological resource in higher education

Resilience, a key aspect of positive adaptation to adversity, is crucial for students navigating academic stress (Aburn et al., 2016; Luthar et al., 2000). Defined as the ability to maintain positive adjustment despite challenges (Luthar et al., 2000), resilience includes both exposure to adversity and effective coping, enabling students to manage academic pressures like demanding coursework and examinations (Aburn et al., 2016; Herrman et al., 2011). To ensure consistency in interpretation, this study specifically operationalizes resilience as the ability to recover from stress—measured by the Brief Resilience Scale (BRS)—which isolates the “recovery” aspect of the construct from the “perseverance” aspect of grit (Smith et al., 2008). Contemporary structural modeling has unveiled that academic resilience is a multi-factorial construct comprising positive individual characteristics as well as external pillars like family, teacher, and peer support (Duan et al., 2024). This multi-dimensional structure confirms that resilience is not an isolated internal trait but a dynamic intersection of personal grit and social connectedness.

In demanding academic settings, resilience is vital for overcoming difficulties and managing stressors. Resilient students consistently show a greater capacity to navigate academic rigors, experiencing less burnout and anxiety than less resilient peers (García-Izquierdo et al., 2018; Ríos-Risquez et al., 2016; Wang et al., 2022). Furthermore, academic buoyancy—a construct closely related to everyday resilience—has been shown to mediate the relationship between social support and reduced burnout (Li et al., 2025). This suggests that the “mechanism” of resilience identified in our model serves a protective function by sequentially reducing negative affective states, thereby clearing a path for optimism (Derakhshan and Fathi, 2025). Resilience also significantly fosters academic success. Research links resilience to better academic performance across education levels (Ayala and Manzano, 2018; Novotný and Kreménková, 2016; Rodríguez-Fernández et al., 2018). The theoretical interrelationship between these variables positions resilience as a transformative process: while grit provides the “proactive engine” of persistence and social connectedness provides the “external fuel” of support, resilience acts as the “internal mechanism” that processes these inputs to maintain emotional stability during disruptions. This explains why resilience is modeled as a mediator; it is the active process of utilizing internal tenacity and external resources to return to a baseline of functioning, which in turn fosters a generalized expectation of positive outcomes—optimism (Masten and Cicchetti, 2016; Sagi et al., 2021).

The relationship between resilience and academic grit is noteworthy, particularly how perseverance builds resilience (Calo et al., 2022). While grit is sustained passion and perseverance for long-term goals (Duckworth et al., 2007), resilience is specifically the capacity to recover from setbacks. In this model, they are synergistic rather than redundant: grit ensures the student does not give up on the goal, while resilience ensures the student recovers from the emotional toll of the obstacle. Studies in demanding fields like dentistry and nursing highlight their complementarity. Grit and resilience strongly predicted academic success in dental students (Montas et al., 2021), and a positive correlation exists between grit and resilience in nursing students (Meyer et al., 2020). While grit drives consistent effort, resilience enables recovery from academic challenges, synergistically contributing to long-term academic goals.

Furthermore, resilience mediates relationships between psychological constructs like grit, social connectedness, and optimism, acting as a buffer that transforms adversity into growth (Masten and Cicchetti, 2016; Wright et al., 2012). Oriol et al. (2020) found resilience mediates the link between optimism and life satisfaction in adolescents, suggesting resilient individuals maintain optimism despite difficulties. This mediation occurs because resilience prevents the “exhaustion” of psychological resources. By effectively recovering from stress, resilient students protect their cognitive capacity to believe in favorable future results. Empirical evidence suggests that without the mediating effect of resilience, the raw effort of grit might lead to burnout rather than optimism (García-Izquierdo et al., 2018). Sagi et al. (2021) also found resilience mediates the relationship between social connectedness and well-being, indicating socially connected students are more likely to develop resilience, enhancing emotional health and stress coping.

### Optimism as a predictor of academic success and well-being

Optimism, defined as a generalized expectation of positive outcomes even amidst challenges, is a crucial disposition influencing individuals’ approaches to life (Scheier and Carver, 1985). This hopeful outlook reflects a belief in one’s ability to positively influence events and anticipate favorable results across life domains (Carver and Scheier, 2014; Peterson, 2000). Optimism, measured by the Life Orientation Test (LOT), is rooted in a broad belief in positive futures, distinct from hope or self-efficacy, which may involve direct personal control (Carver and Scheier, 2023; Scheier and Carver, 1985). This understanding has driven extensive research into optimism’s implications for academic success, well-being, and coping strategies.

Empirical research robustly demonstrates optimism’s pivotal role in positive academic outcomes. Optimistic students consistently show higher achievement, better coping, and increased motivation across educational settings (Boonen et al., 2014; Rand et al., 2020; Tetzner and Becker, 2018). The reason optimism is the downstream result of grit and social connectedness is rooted in the cognitive reframing that occurs through resilience. When students successfully bounce back (resilience) using their own tenacity (grit) and their community’s support (social connectedness), they strengthen the belief that future challenges will also be surmountable. This reinforces the interrelationship where optimism is both a product of past resilience and a driver of future grit (Pyo et al., 2024).

Furthermore, optimism effectively buffers against academic stress. Optimistic students tend to perceive stressful situations as temporary and manageable, enabling more effective coping mechanisms (Carver and Scheier, 2023; Tan and Tan, 2014). This positive disposition encourages problem-focused coping, mitigating the emotional toll of academic pressures (Carver and Scheier, 2023). This highlights the integrative nature of these four constructs: grit and social connectedness provide the means to survive academic rigors, resilience provides the method for recovery, and optimism provides the motivation to continue the cycle.

The link between optimism and resilience further highlights optimism’s role in managing academic challenges. Resilient students, often optimistic, recover faster from setbacks and remain committed to academic goals (Genç and Arslan, 2021; Gómez Molinero et al., 2018; Hojat et al., 2015; Perera and McIlveen, 2014; Romano et al., 2021a,b). Optimism mitigates stress’s immediate impact and provides emotional resources for sustained engagement and long-term success (Anderson et al., 2018; Saleem et al., 2022). This interplay shows how optimism enhances resilience, fostering both well-being and academic persistence, creating a positive cycle of resilience and achievement.

Moreover, optimism is intrinsically linked to social connectedness, a key factor in student well-being and academic outcomes (Olasupo et al., 2021). Optimistic individuals are generally more adept at forming and maintaining positive social relationships, enriching their academic experiences (Cabras and Mondo, 2018; Phan, 2016; Yıldırım et al., 2021). Research indicates optimistic students build stronger peer bonds and engage more actively in academic communities (Cabras and Mondo, 2018). This social connectedness reinforces optimism, creating a beneficial loop where positive social interactions strengthen resilience and academic performance (Phan, 2016; Sagi et al., 2021). Thus, optimism, resilience, and social connectedness form an interconnected network, each enhancing student well-being and academic success.

### This study

Fostering student well-being and academic success requires a comprehensive understanding of the psychological resources that enable thriving. Research identifies academic grit, social connectedness, resilience, and optimism as fundamental correlates of positive outcomes. Academic grit, defined by sustained perseverance toward long-term goals (Duckworth et al., 2007), provides the tenacity to navigate academic challenges and serves as a primary foundation for resilience (Clark and Malecki, 2019; Tang et al., 2019). Optimism, characterized by generalized positive outcome expectancies (Scheier and Carver, 1985), is associated with enhanced achievement, effective coping, and overall well-being (Rand et al., 2020; Tetzner and Becker, 2018). Furthermore, social connectedness provides the essential emotional and practical support required to manage the unique pressures of higher education (Allen et al., 2008).

Building on this evidence, the current study investigates the interrelations of these constructs as a context-situated process within the Chinese university setting. While the cross-sectional nature of our data precludes definitive causal claims, we proposed a hypothesized directional model grounded in established psychological frameworks. Drawing on the Conservation of Resources (COR) theory (Hobfoll, 1989), we posit that personal and social assets (grit and connectedness) serve as foundational resources that precede functional adaptive capacities like resilience. Furthermore, based on the Broaden-and-Build theory (Fredrickson, 2001), we suggest that the capacity for recovery (resilience) provides the emotional-cognitive foundation for maintaining positive future expectations (optimism).

Based on this theoretical scaffolding, we hypothesized that: (1) academic grit and (2) social connectedness would be positively associated with resilience. We further hypothesized that: (3) academic grit and (4) social connectedness would be positively associated with optimism. Finally, we hypothesized that (5) resilience would function as an intervening variable, statistically accounting for the associations between grit, social connectedness, and optimism. These hypotheses position resilience as a potential mechanism through which internal tenacity and external social bonds relate to positive future expectations.

To examine these relationships, we employed an explanatory sequential mixed-methods design. The quantitative phase used Structural Equation Modeling (SEM) to examine direct and indirect statistical associations. The subsequent qualitative phase utilized student diaries to provide processual insights into the lived experiences of these constructs. This qualitative exploration is intended to explain and extend the statistical findings, illuminating the self-reflective meaning-making students use to cultivate resilience and optimism.

## Methods and materials

This study utilized an explanatory sequential mixed-methods design (Creswell and Plano Clark, 2018). This design commenced with quantitative data collection and analysis to identify broad patterns of association, followed by a qualitative phase intended to explain and extend these statistical findings. Rather than simply validating causal pathways, the qualitative phase sought to illuminate the lived experiences and meaning-making processes that underlie the statistical model. Quantitative data were initially gathered and analyzed to test the hypothesized structural model. Subsequently, qualitative data were collected from a purposefully selected subset of participants through diary entries to provide a processual explanation of how students internalize grit and utilize social support to cultivate resilience.

### Participants

This study included 372 undergraduate students (183 male, 189 female) aged 18–25 years (*M* = 20.3, SD = 1.85) from two large comprehensive “Double First-Class” (Tier 1) universities in eastern China. Participants represented diverse academic disciplines: approximately 35% engineering, 30% business, 20% humanities, and 15% natural sciences, mirroring the universities’ program distribution. Inclusion criteria required participants to be full-time undergraduates (years 1–4) with no self-reported history of clinical mental health disorders that might confound well-being metrics. Students were excluded if they were currently on academic probation to ensure the sample reflected the standard “demanding” academic environment rather than an outlier state of academic crisis.

Recruitment used a non-probability convenience sampling strategy via email lists and lecture hall announcements. To minimize self-selection bias, the recruitment materials framed the study as a general survey on “Student Academic Experiences” rather than focusing on high-performing or “gritty” students. The recruitment period lasted exactly 21 days during the mid-semester period (Patton, 2015) to capture a stable window of academic activity. While this approach allowed for a robust sample size within the practical constraints of an exploratory mixed-methods design, it is important to note that the sample primarily consists of students from academically selective provincial-level “key” high schools and urban/semi-urban backgrounds.

For the qualitative phase, an intensity sampling strategy was employed (Patton, 2015). We selected a subset of 40 participants who scored ±1 standard deviation from the mean on the Academic Grit and Resilience scales in the quantitative phase. This purposive selection aimed to capture “information-rich” cases that clearly manifested the constructs under investigation. Socioeconomic data was not precisely collected for anonymity, but estimations based on typical enrollment suggest 65–70% were from middle-class backgrounds, with the remainder from working-class (25–30%) and upper-middle class families (approximately 5%). Participation was voluntary, and all participants provided informed consent. The study received IRB ethical approval from Qilu Medical University. Demographic data (age, gender, major, year, GPA) was collected for subgroup analysis. Further context indicates approximately 70% lived in on-campus dormitories. All personal identifiers were anonymized, with data securely stored and diary entries redacted to protect privacy.

### Instruments

All scales used in this study were originally developed in English. To ensure linguistic and conceptual equivalence for the Chinese context, a translation and back-translation procedure was implemented by two independent bilingual researchers (Applied Linguistics specialists). Discrepancies were resolved through committee approach. The full set of items for all four instruments is provided in the Appendix.

#### Social connectedness

The Social Connectedness Scale-Revised (SCS-R; Lee and Robbins, 1995) was employed to assess participants’ sense of belonging and emotional connection within their social networks. This 20-item scale includes both positively and negatively worded statements, which participants rated on a 6-point Likert scale (1 = strongly disagree to 6 = strongly agree). Individual responses were averaged across the 20 items to produce a dimension-level mean ranging from 1 to 6. Higher scores on this scale suggest a stronger feeling of social connectedness and a greater sense of intimacy in relationships. For the SCS-R, CFA results in this study indicated robust construct validity: χ^2^/df = 2.89, CFI = 0.93, TLI = 0.92, RMSEA = 0.073 (90% CI [0.065, 0.080]), SRMR = 0.051. The SCS-R also demonstrated good reliability in this study, with a Cronbach’s alpha of 0.89.

#### Optimism

Optimism was measured using the Life Orientation Test-Revised (LOT-R; Scheier and Carver, 1985), an 8-item scale that evaluates participants’ general expectations for positive outcomes in life. Respondents rated their agreement with items such as “In uncertain times, I usually expect the best” on a 7-point Likert scale (1 = strongly disagree to 7 = strongly agree). The scores were averaged to generate a mean score per item, ranging from 1 to 7. Higher scores indicate a more optimistic outlook, while lower scores suggest less positive future expectations. Evidence for acceptable construct validity of the LOT-R in this sample was provided by CFA, with fit indices as follows: χ^2^/df = 2.21, CFI = 0.96, TLI = 0.95, RMSEA = 0.059 (90% CI [0.048, 0.070]), SRMR = 0.038. In the present study, the LOT-R showed good internal consistency, with a Cronbach’s alpha of 0.85.

#### Academic grit

Academic Grit was assessed using the Academic Grit Questionnaire developed by Clark (2017). This 10-item scale measures the perseverance and passion of students in their academic endeavors. Functionally, this instrument was selected to capture the proactive “stamina” of participants—their ability to maintain interest and effort toward graduation over years, independent of specific setbacks. Participants rated each item on a 5-point Likert scale, ranging from 1 (strongly disagree) to 5 (strongly agree). Like the other sclaes, dimension-level means were utilized for analysis, with scores ranging from 1 to 5. The scale demonstrated strong reliability in previous studies, with Cronbach’s alpha reported as 0.79 (Clark, 2017). Additionally, exploratory factor analysis indicated that all items loaded significantly above 0.50, confirming the scale’s validity for measuring grit in academic contexts. CFA in the current study corroborated the construct validity of the Academic Grit Questionnaire, yielding these fit statistics: χ^2^/df = 3.15, CFI = 0.92, TLI = 0.91, RMSEA = 0.078 (90% CI [0.070, 0.085]), SRMR = 0.055. For the current sample, the Academic Grit Questionnaire demonstrated good internal consistency, with a Cronbach’s alpha of 0.87.

#### Resilience

Participants’ resilience was evaluated using the Brief Resilience Scale (BRS; Smith et al., 2008). Unlike the grit scale, which measures proactive persistence, the BRS was specifically chosen to operationalize the reactive “recovery” or “elasticity” of students. The scale consists of six items, such as “I tend to bounce back quickly after hard times,” with responses provided on a 5-point Likert scale, ranging from 1 (strongly disagree) to 5 (strongly agree). To reflect the actual level of the variable, an average score was calculated by dividing the total sum by the number of items. Consequently, final scores ranged from 1 to 5, with higher scores reflecting stronger resilience. The construct validity of the BRS in this study was supported by CFA, which yielded the following fit indices: χ^2^/df = 2.56, CFI = 0.95, TLI = 0.94, RMSEA = 0.068 (90% CI [0.055, 0.081]), SRMR = 0.042. In the current sample, the BRS demonstrated good internal consistency, with a Cronbach’s alpha of 0.82.

### Student diaries

For the qualitative component, the subset of 40 participants (20 males, 20 females) kept personal diaries over a two-week period. To ensure data quality and minimize “reflection fatigue,” diaries were submitted via a password-protected Qualtrics portal on a bi-weekly schedule (Tuesday and Friday). Automated email reminders were sent 24 h prior to each submission deadline. The diary entries were structured around three semi-structured open-ended prompts:

1. “Describe a recent academic challenge you faced (e.g., a difficult assignment, exam stress, or feedback). How did you respond to it?”
2. “How did your connections with others (peers, faculty, or family) help you during this challenge?”
3. “What thoughts and feelings did you experience regarding your future academic success during this time?”

To facilitate depth, a minimum word count of 200 words per entry was required, though the average entry length was 342 words. The diary method was chosen to facilitate the collection of real-time, introspective data, offering deeper insights into participants’ immediate processes of navigating academic challenges. Diaries were anonymized before being analyzed, ensuring participant privacy and confidentiality. The qualitative data from the diaries were subjected to thematic analysis to identify key patterns and recurring themes related to resilience, social connectedness, and optimism. While the prompts provided a structure to guide participants’ reflections toward the core constructs of the study, it is acknowledged that the directive nature of these prompts may have potentially influenced the scope and depth of the qualitative data. Future qualitative investigations could consider employing more open-ended and less structured approaches, such as unstructured journaling or in-depth interviews, to further explore the nuances of students’ experiences in this domain.

### Procedure

Recruitment began with announcements via university mailing lists and in-class instructor announcements at participating universities. Interested students were directed to an online registration form on Qualtrics, where they registered and provided contact details. Recruitment lasted approximately three weeks, with weekly reminders to maximize participation. After registration, participants received a detailed explanation of the study’s objectives, methods, and ethical considerations via email, along with a digital consent form. An optional virtual information session was offered to address questions.

Data collection spanned five weeks. The quantitative phase, measuring Academic Grit, Social Connectedness, Resilience, and Optimism via self-report questionnaires, occurred during the first two weeks. Participants received a secure link to the online survey via email, which they completed at their convenience in one sitting (approximately 25–30 min) to minimize distractions and ensure response accuracy. Honest answers were requested, and participants were informed of no right or wrong answers. To incentivize participation, students could optionally enter a raffle to win one of five 50 gift cards.

The qualitative phase started immediately after quantitative data collection. From the 372 participants, a subset of 40 students was selected based on availability and willingness to engage in the diary component. These students were individually emailed and asked to maintain a personal diary for two weeks. Diary submissions were online, via Qualtrics, ensuring data collection consistency. Participants were instructed to submit at least two entries weekly, but encouraged to write more frequently during significant academic challenges. Each diary entry was to be reflective and descriptive, focusing on lived experiences of academic grit, social connectedness, resilience, and optimism. A mid-point check-in after the first week ensured compliance and addressed technical or prompt-related questions.

Throughout the study, participant privacy and anonymity were protected. Unique alphanumeric codes were assigned to each participant to link quantitative survey data with qualitative diary entries while removing all personally identifiable information (PII) from the final analysis dataset. Personal data and diary entries were stored on encrypted servers, and only anonymized datasets were analyzed. Following diary submissions, participants received a debriefing email outlining research next steps and thanking them for their contributions.

## Data analysis

Data analysis involved two phases: quantitative data analysis followed by qualitative diary entry analysis. For quantitative analysis, self-report questionnaire responses were exported from Qualtrics and cleaned for accuracy. Data screening checked for missing data and outliers. Missing data (less than 5%) were handled using multiple imputation (Rubin, 2004) to maintain statistical power and validity. Descriptive statistics (means, standard deviations, correlations) were generated to provide an initial data overview.

Structural Equation Modeling (SEM) in AMOS version 26 was the primary quantitative analysis method, chosen for its ability to examine complex relationships between variables while accounting for measurement error (Byrne, 2016). All models were estimated using the Maximum Likelihood (ML) method. Confirmatory factor analysis (CFA) was first conducted to validate the measurement model, ensuring scales for Academic Grit, Social Connectedness, Resilience, and Optimism reliably measured their constructs. Model fit was assessed using standard criteria: Comparative Fit Index (CFI), Tucker-Lewis Index (TLI), Root Mean Square Error of Approximation (RMSEA), and Standardized Root Mean Square Residual (SRMR). Following the recommendations of Hu and Bentler (1999), CFI and TLI values > 0.90 and SRMR < 0.08 were used to indicate acceptable fit. Regarding RMSEA, while values < 0.06 suggest a close fit, values up to 0.08 are considered indicative of an adequate or reasonable error of approximation (Browne and Cudeck, 1992; MacCallum et al., 1996).

**Figure 1:**
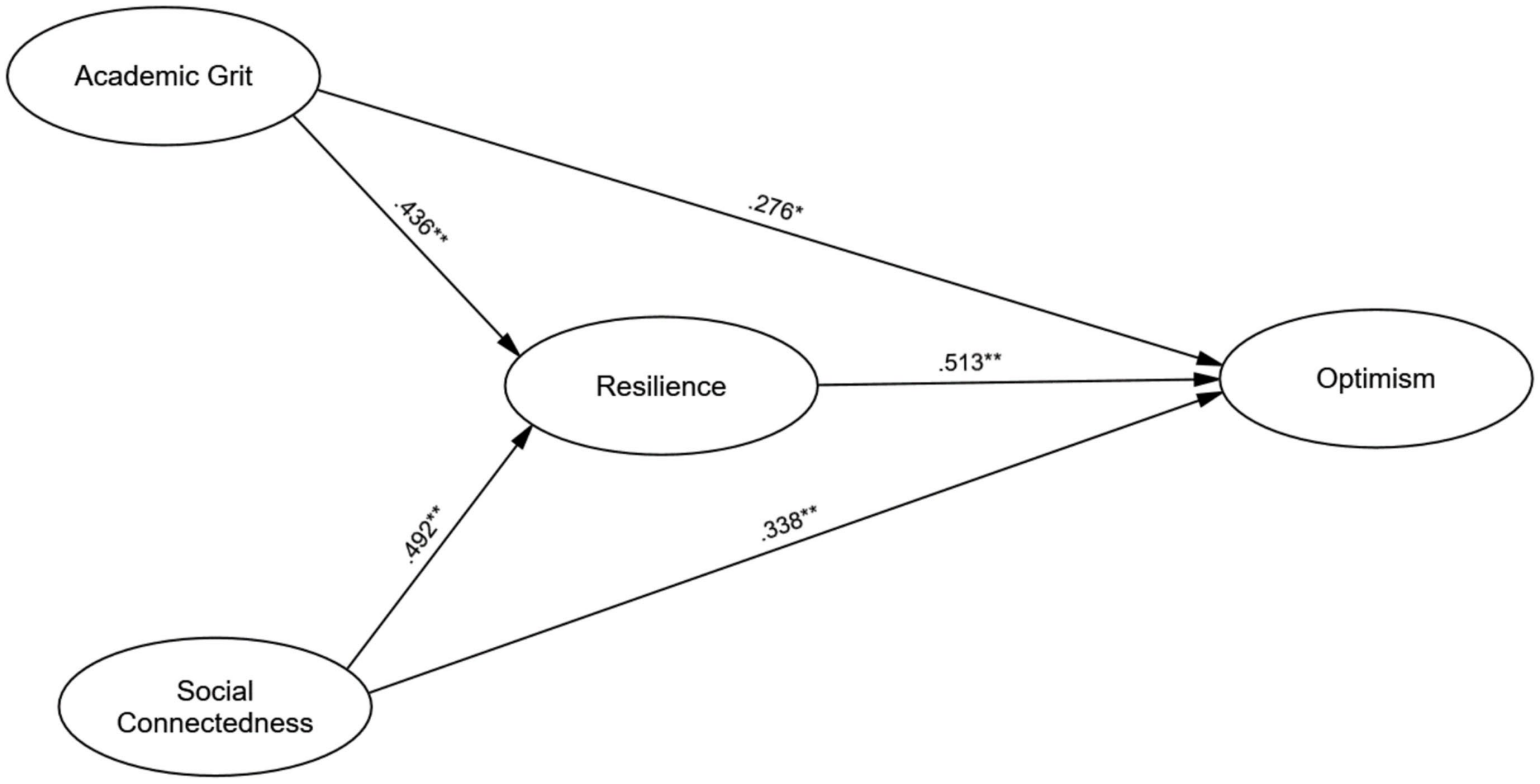
Structural equation model illustrating the relationships between academic grit, social connectedness, resilience, and optimism.

The hypothesized structural model followed a theory-driven framework positioning individual dispositions (Academic Grit) and social resources (Social Connectedness) as antecedents to adaptive processes (Resilience), which ultimately foster positive future expectations (Optimism). The paths from grit and social connectedness to resilience were grounded in Conservation of Resources (COR) theory (Hobfoll, 1989), which posits that personal and social assets function as “resource caravans” to build functional capacities. Furthermore, the directional link from resilience to optimism was justified by the Broaden-and-Build theory (Fredrickson, 2001), suggesting that recovery from adversity expands a student’s cognitive and emotional repertoire, leading to a more optimistic orientation. The model examined both the direct effects of grit and social connectedness on optimism and their indirect effects mediated by resilience. By treating resilience as a mediator, the study tests the specific mechanism through which personal tenacity and structural supports are converted into a stable optimistic outlook. Finally, bootstrapping with 5,000 resamples (Hayes, 2018) was employed to test the significance of these indirect effects and assess the model’s overall explanatory power (Figure 1).

Following the structural analysis, we performed further diagnostic tests to ensure model integrity. First, modification indices (MI) were examined to identify potential localized areas of misfit and theoretically justifiable model refinements. Second, multicollinearity was assessed using the Variance Inflation Factor (VIF) and Tolerance values for all predictor variables. All predictor variables fell within acceptable ranges (VIF < 5, Tolerance > 0.2), ruling out excessive multicollinearity that could otherwise inflate standard errors and bias path coefficients (Hair et al., 2017).

To maintain interpretive rigor, a codebook was established in NVivo 12, incorporating clear definitions, inclusion/exclusion criteria, and representative data extracts (MacQueen et al., 1998). We employed a hybrid inductive-deductive approach (Fereday and Muir-Cochrane, 2006), beginning with data familiarization and progressing through open coding to identify significant statements. Subsequent axial coding grouped these into broader themes. Thematic saturation was achieved after 35 diaries, as the final five entries yielded no novel codes. Two independent researchers analyzed the data, achieving an 88% inter-coder agreement, with the remaining 12% resolved through negotiated agreement to refine definitions. Final themes included “perseverance in academic adversity,” “social support as emotional buffer,” and “optimism as forward-looking mindset.” This audit-ready framework ensures that findings are grounded in the raw data rather than researcher bias. The analysis concluded with a “meta-inference” synthesis, where quantitative paths were mapped against qualitative narratives using a joint display (Creswell and Plano Clark, 2018). Rather than merely seeking confirmation, this integration identified areas of expansion or divergence, explaining the psychological contingencies underlying the statistical model.

## Findings

### Quantitative results

#### Descriptive statistics and correlations

To begin, descriptive statistics were calculated using item-level means for all key variables. These values, presented in Table 1, offer a clearer view of where participants fall on the respective Likert scales. For academic grit, the mean was 3.76 (*SD* = 0.62) on a 5-point scale, indicating that participants exhibited moderate to high levels of grit. Social connectedness had a mean of 4.92 (*SD* = 0.77) on a 6-point scale, reflecting relatively strong social ties. The mean for resilience was 3.73 (*SD* = 0.69) on a 5-point scale, demonstrating a moderate ability to recover from stress. Meanwhile, optimism had a mean of 5.52 (*SD* = 0.87) on a 7-point scale, indicating a generally optimistic outlook among participants.

**Table 1:** Descriptive statistics (item-level means).

Variable	Mean	SD	Min	Max
Academic Grit	3.76	0.62	1.00	5.00
Social connectedness	4.92	0.77	2.25	6.00
Resilience	3.73	0.69	1.67	5.00
Optimism	5.52	0.87	3.13	7.00

Correlational analyses (Table 2) revealed significant positive associations between all variables. To check for multicollinearity before proceeding to the structural model, we examined the VIF and Tolerance values. The VIF values for Academic Grit (1.28), Social Connectedness (1.35), and Resilience (1.44) were all well below the conservative threshold of 5.0, while Tolerance values ranged from 0.69 to 0.78. These diagnostics confirm that multicollinearity did not pose a significant threat to the validity of the structural paths. Even after converting to item-level means, academic grit remained moderately correlated with social connectedness (*r* = 0.34, *p* < 0.001), resilience (*r* = 0.45, *p* < 0.001), and optimism (*r* = 0.38, *p* < 0.001). Similarly, social connectedness was strongly correlated with resilience (*r* = 0.56, *p* < 0.001) and moderately with optimism (*r* = 0.42, *p* < 0.001). The strongest relationship was observed between resilience and optimism (*r* = 0.58, *p* < 0.001).

**Table 2:** Correlations between variables.

Variable	Academic grit	Social connectedness	Resilience	Optimism
Academic grit	1.00	0.34***	0.45***	0.38***
Social connectedness	0.34***	1.00	0.56***	0.42***
Resilience	0.45***	0.56***	1.00	0.58***
Optimism	0.38***	0.42***	0.58***	1.00

***p < 0.001.

#### Measurement model

Confirmatory Factor Analysis (CFA) assessed the measurement model, which included four latent variables: academic grit, social connectedness, resilience, and optimism. CFA results (Table 3) indicated adequate model fit, with acceptable values for key indices: χ^2^(460) = 792.45, *p* < 0.001; CFI = 0.92; TLI = 0.91; RMSEA = 0.065 (90% CI [0.060, 0.071]); and SRMR = 0.049. Although the RMSEA slightly exceeds the strict 0.06 criterion, it remains well within the 0.08 threshold generally accepted for adequate model fit (Browne and Cudeck, 1992).

**Table 3:** Confirmatory factor analysis results.

Latent variable	Indicator	Factor loading	SE	*p*-value
Academic grit	Grit1	0.82	0.03	<0.001
	Grit2	0.75	0.04	<0.001
	Grit3	0.79	0.03	<0.001
	Grit4	0.85	0.02	<0.001
	Grit5	0.71	0.04	<0.001
Social connectedness	SC1	0.68	0.05	<0.001
	SC2	0.81	0.04	<0.001
	SC3	0.77	0.04	<0.001
	SC4	0.70	0.04	<0.001
	SC5	0.82	0.03	<0.001
Resilience	Res1	0.86	0.02	<0.001
	Res2	0.72	0.03	<0.001
	Res3	0.75	0.03	<0.001
	Res4	0.80	0.02	<0.001
	Res5	0.83	0.03	<0.001
	Res6	0.78	0.03	<0.001
Optimism	Opt1	0.73	0.04	<0.001
	Opt2	0.88	0.02	<0.001
	Opt3	0.75	0.03	<0.001
	Opt4	0.70	0.03	<0.001
	Opt5	0.81	0.02	<0.001
	Opt6	0.77	0.03	<0.001
	Opt7	0.73	0.04	<0.001
	Opt8	0.87	0.02	<0.001

Table 3 shows all factor loadings were significant (*p* < 0.001), ranging from 0.67 to 0.88, indicating observed variables reliably measured their latent constructs. For example, academic grit factor loadings ranged from 0.71 to 0.85, and resilience loadings ranged from 0.72 to 0.86. Social connectedness and optimism also showed robust factor loadings, ranging from 0.68 to 0.88, further confirming the measurement model’s validity.

To further evaluate the psychometric properties of the instruments, composite reliability (CR) and average variance extracted (AVE) were calculated (Table 4). Convergent validity was confirmed as all CR values exceeded the 0.70 threshold and all AVE values were above the recommended 0.50 level (Fornell and Larcker, 1981). Specifically, academic grit showed a CR of 0.84 and an AVE of 0.61. Social connectedness yielded a CR of 0.82 and an AVE of 0.58. Resilience and optimism also demonstrated strong convergent validity, with CR values of 0.86 and 0.88 and AVE values of 0.64 and 0.62, respectively. These results indicate that the latent constructs explain a substantial portion of the variance in their respective indicators, ensuring the reliability and validity of the measurement model across the sample.

**Table 4:** Reliability and convergent validity results.

Latent variable	Composite reliability	Average variance extracted (AVE)
Academic grit	0.84	0.61
Social connectedness	0.82	0.58
Resilience	0.86	0.64
Optimism	0.88	0.62

#### Structural model

Following confirmatory factor analysis, the hypothesized structural model was tested using SEM to explore direct and indirect effects of academic grit and social connectedness on optimism, with resilience as a mediator. An initial assessment of the structural model suggested a marginal fit; however, a re-examination of the modification indices revealed high covariance between the error terms of two indicators within the social connectedness scale (SC2 and SC5), likely due to overlapping item content regarding peer support. After specifying a correlation between these error terms to account for this shared systematic variance, the final SEM analysis demonstrated satisfactory data fit: χ^2^(322) = 845.23, *p* < 0.001; CFI = 0.91; TLI = 0.90; RMSEA = 0.070 (90% CI [0.065, 0.076]); and SRMR = 0.055. While the RMSEA of 0.070 is above the 0.06 “close fit” threshold proposed by Hu and Bentler (1999), it is considered an acceptable value for a complex structural model, representing a reasonable fit to the data (MacCallum et al., 1996). The robust performance of the other fit indices—particularly the SRMR, which is well below the 0.08 limit—further supports the overall adequacy of the structural model.

Analysis of direct effects (Table 5) revealed that academic grit had a significant positive effect on resilience (*β* = 0.436, *SE* = 0.06, *p* < 0.001), as did social connectedness on resilience (*β* = 0.492, *SE* = 0.05, *p* < 0.001). Academic grit (*β* = 0.276, *SE* = 0.07, *p* < 0.001) and social connectedness (*β* = 0.338, *SE* = 0.06, *p* < 0.001) also had significant direct effects on optimism. Resilience showed the strongest direct effect on optimism (*β* = 0.513, *SE* = 0.04, *p* < 0.001), confirming its crucial role in fostering optimism.

**Table 5:** Direct effects.

Path	*β*	SE	*p*-value
Academic grit → resilience	0.436	0.06	<0.001
Social connectedness → resilience	0.492	0.05	<0.001
Academic grit → optimism	0.276	0.07	<0.001
Social connectedness → optimism	0.338	0.06	<0.001
Resilience → optimism	0.513	0.04	<0.001

Mediation analysis (Table 6) indicated that resilience functioned as a significant intervening variable in the associations between both academic grit and social connectedness with optimism. The indirect association of academic grit on optimism via resilience was significant (*β* = 0.223, *SE* = 0.04, *p* < 0.001, 95% CI [0.15, 0.29]), as was the indirect effect of social connectedness on optimism via resilience (*β* = 0.252, *SE* = 0.03, *p* < 0.001, 95% CI [0.20, 0.32]). Bootstrapping confirmed the significance of these indirect effects.

**Table 6:** Indirect associations (intervening paths).

Path	*β*	SE	*p*-value	95% CI (Lower, Upper)
Academic grit → resilience → optimism	0.223	0.04	<0.001	[0.15, 0.29]
Social connectedness → resilience → optimism	0.252	0.03	<0.001	[0.20, 0.32]

Total effects (Table 7) showed that academic grit had a total effect on optimism of β = 0.499 (*SE* = 0.06, *p* < 0.001), and social connectedness had a total effect on optimism of β = 0.590 (*SE* = 0.05, *p* < 0.001). These total effects indicate substantial overall contributions from both predictors to optimistic outlook, with resilience playing a pivotal mediating role.

**Table 7:** Total effects.

Path	*β*	SE	*p*-value
Academic grit → optimism	0.499	0.06	<0.001
Social connectedness → optimism	0.590	0.05	<0.001

The final structural model explained 44% of the variance in resilience (*R*^2^ = 0.44) and 57% of the variance in optimism (*R*^2^ = 0.57) (Figure 1), suggesting that academic grit, social connectedness, and resilience robustly explain individual differences in optimism among university students.

## Qualitative results

While the *SEM* results provided statistical evidence that resilience intervenes in the associations between grit, social connectedness, and optimism, the qualitative data elucidated the internal dialogue facilitating this transition. The thematic analysis, grounded in the iterative codebook development described above, reveals a dynamic, non-linear process that extends beyond the stable associations found in the statistical model. The statistical model identifies stable associations, whereas the diary entries reveal a dynamic, non-linear process. For example, although the *SEM* indicates a strong path from social connectedness to resilience, the qualitative findings explain that this is not a passive receipt of support, but an active “social navigation” where students selectively filter their networks to meet specific emotional or academic needs. Furthermore, while the quantitative model suggests grit predicts resilience, the qualitative narratives explain that this occurs through a “vulnerability–strength” dialectic in which students consciously choose to persevere through doubt—a meaning-making process that static survey items cannot fully capture.

The qualitative data consisted of 80 diary entries submitted by a subset of 40 participants (20 males, 20 females), with each participant providing two entries over a two-week period. These entries, ranging from 200 to 450 words, offered a rich source of data, revealing participants’ nuanced experiences with academic challenges, social connectedness, resilience, and optimism. The thematic analysis of these entries resulted in three core themes: Perceptions of Academic Vulnerability and Strength, Adaptive Social Navigation in Academic Life, and Resilience as a Self-Reflective Journey. These themes are intended to provide a process-oriented explanation of the statistical associations reported in the quantitative phase.

### Theme 1: perceptions of academic vulnerability and strength: acknowledging doubt, discovering capacity

This theme highlights the tension between feelings of vulnerability and emerging strength as participants grappled with academic pressures. Participants frequently described moments of doubt and insecurity, particularly in relation to high-stakes assessments like exams or major assignments. For instance, Participant 12 (Male, Business major) expressed feeling overwhelmed during midterms:

“During the week of midterms, I constantly felt like I wasn’t good enough. Every subject seemed harder than the last, and no matter how much I studied, I doubted whether I would be able to succeed. It wasn’t just about the grades; it was this deeper fear that I wasn’t cut out for university at all. I started questioning my choices, wondering if I had overestimated my abilities. It was like I was running in circles without getting anywhere, and the finish line kept moving further away.”

This sentiment was echoed by several participants who described a pervasive sense of academic inadequacy when faced with demanding tasks. Yet, intertwined with this vulnerability was the recognition of inner strength, often realized after overcoming academic hurdles. For example, Participant 33 (Female, Engineering major) reflected on this duality after completing a difficult essay:

“After I finished my final essay, I realized that, although it was tough and I doubted myself throughout the entire process, questioning if my arguments were strong enough and if I truly understood the material, I had managed to stay focused and complete it. That gave me a sense of accomplishment and made me realize that I am stronger than I thought. It wasn’t just about getting a good grade; it was about proving to myself that I can persevere even when I feel completely overwhelmed. This experience made me feel more confident in tackling future challenges, knowing I have this inner reserve of strength I can tap into.”

Participants’ experiences of oscillating between doubt and empowerment demonstrate the complex emotional landscape of academic life, where feelings of weakness can coexist with moments of personal growth and resilience. This theme directly supports the quantitative finding of a significant positive relationship between Academic Grit and Resilience (*β* = 0.436, *p* < 0.001). The diary entries illuminate *how* this relationship manifests in students’ lived experiences. The qualitative data reveals that academic grit, characterized by perseverance and sustained effort, is not simply about achieving high grades, but also about navigating and overcoming these inherent feelings of vulnerability. By pushing through moments of doubt and insecurity (as illustrated by Participant 12), students actively build their resilience, recognizing their capacity for sustained effort and achievement (as shown in Participant 33). The qualitative narratives thus enrich the statistical finding by demonstrating the *process* through which grit contributes to resilience: by confronting and overcoming perceived academic weaknesses. Many students acknowledged that these difficult experiences ultimately contributed to their development of academic grit, as they learned to push through initial feelings of vulnerability and recognize their capacity for sustained effort and achievement.

### Theme 2: adaptive social navigation in academic life: strategic support for varied needs

Social connectedness emerged as a central adaptive mechanism for managing academic stress. Participants consistently referred to their social networks as essential for maintaining emotional balance and navigating academic pressures. For example, Participant 9 (Female, Psychology major) described the relief that came from sharing her concerns with peers:

“I was really stressed out about the group project, but talking it over with my classmates helped a lot. We were all in the same boat, so just knowing that I wasn’t the only one feeling this way was comforting. It wasn’t just about sharing the workload; it was the emotional validation of knowing I wasn’t alone in struggling. We decided to meet up more often to share ideas and make sure everyone was on the same page, but more importantly, to just support each other through the stress. Knowing they understood made me feel less isolated and more capable of handling the pressure.”

While peer support played a vital role, participants also demonstrated agency in seeking out different types of social connections based on the nature of the challenge they faced—a process referred to as adaptive social navigation. Many participants reported strategically engaging specific members of their social circles depending on their needs. For example, Participant 21 (Male, Economics major) articulated this flexibility:

“When I feel lost academically, I usually ask my study group for help. They always have good tips on how to tackle assignments. They understand the course content and can explain things in a way that makes sense to me. But when I’m really stressed and anxious about my performance overall, I talk to my sister because she’s always been there for me emotionally. She doesn’t necessarily understand econometrics, but she knows how to calm me down and remind me of my strengths. It’s like I have different people for different kinds of support – academic and emotional.”

This active and selective engagement with social resources underscores the dynamic nature of social support in academic settings. Rather than passively relying on their networks, participants demonstrated an ability to navigate different forms of social support, drawing on peers for practical academic help and turning to family or close friends for emotional reassurance. The qualitative narratives enrich the statistical finding by demonstrating that social connectedness is not merely a static “resource,” but a lived practice of strategic filtration where students match specific social assets to specific academic stressors.

This theme directly expands upon the SEM finding that social connectedness has significant direct effects on both resilience (*β* = 0.492, *p* < 0.001) and optimism (*β* = 0.338, *p* < 0.001). The diary entries illuminate the functional distinction between these traits: students described “grit” as the proactive decision to keep attending classes and maintaining focus despite chronic exhaustion, whereas they described “resilience” as the specific, reactive emotional recovery required after a concrete setback, such as receiving a failing grade on a midterm. The qualitative narratives thus enrich the statistical finding by demonstrating that grit is the behavioral framework—the unwavering commitment to the degree—that makes the resilient recovery (bouncing back from a specific failure) a necessity rather than an option. By pushing through moments of doubt and insecurity (as illustrated by Participant 12), students actively build their resilience, recognizing their capacity for sustained effort and achievement (as shown in Participant 33). The qualitative data reveals that academic grit is not simply about achieving high grades, but about navigating and overcoming these inherent feelings of vulnerability. Many students acknowledged that these difficult experiences ultimately contributed to their development of academic grit, as they learned to push through initial feelings of vulnerability and recognize their capacity for sustained effort.

### Theme 3: resilience as a self-reflective journey: learning, adapting, and growing from setbacks

Resilience emerged as a key theme, not as a static trait but as a process that participants actively cultivated through self-reflection and introspection. This theme explains the ‘how’ behind the statistical mediation found in the SEM. While the SEM identifies that resilience accounts for the variance in optimism, the diaries reveal the underlying cognitive reframing necessary for this association to exist. Many participants described their resilience as something that developed over time, particularly as they learned from previous academic setbacks. This theme aligns with the notion of resilience as a dynamic and evolving capacity, shaped by experiences and cognitive reframing. Participant 7 (Female, History major) captured this evolving understanding of resilience, noting how her mindset shifted over the course of the semester:

“At the beginning of the semester, I would get really anxious whenever I got a bad grade. It felt like a personal failure, and I would spiral into negative thoughts about my abilities. But after a few setbacks, I started to change my approach, consciously trying to reframe my thinking. I realized that instead of focusing on the failure, I needed to focus on what I could do better next time. It wasn’t easy at first, but it’s helped me build resilience. Now, when I face a setback, my first reaction is to analyze what happened and plan my next steps, rather than just getting discouraged. It’s a shift from emotional reactivity to proactive problem-solving.”

This narrative explains that the intervening role of resilience is driven by a shift from emotional reactivity to proactive problem-solving. This reflective process was common across participants, who often described resilience as something they actively worked on, refining their coping strategies and learning from both successes and failures. Participant 18 (Male, Computer Science major) eloquently summarized this perspective:

“Resilience isn’t just about bouncing back; it’s about figuring out why you fell in the first place and making sure you don’t fall the same way again. It’s like each setback is a learning opportunity… This reflection is crucial; it’s how you actually grow stronger and develop true resilience, not just temporary coping mechanisms.”

Participants emphasized that resilience required not only perseverance but also a willingness to engage in self-reflection and adjust their behaviors. Many highlighted specific strategies they had developed over time, such as improved time management, self-compassion, and cognitive reframing, which helped them to better cope with academic stressors. For these students, resilience was a journey of personal growth, shaped by trial and error. This theme provides crucial qualitative explanation for the intervening role of resilience in the associations between academic grit and social connectedness with optimism. The significant indirect associations (Academic Grit → Resilience → Optimism: *β* = 0.223, *p* < 0.001; Social Connectedness → Resilience → Optimism: *β* = 0.252, *p* < 0.001) are not merely statistical pathways, but reflect a lived process of self-reflection and adaptation. The diary entries explain the process by which resilience acts as an intervening variable. Participant 7’s narrative illustrates the shift from reactive anxiety to proactive problem-solving, a key aspect of resilience development that statistically accounts for the association between setbacks and optimism. Participant 18 emphasizes the self-reflective component of resilience that allows students to convert negative experiences into opportunities for growth. The qualitative data, therefore, explains the psychological mechanics through which resilience intervenes in the effects of grit and social connectedness on optimism, showing it as an active, evolving process of cognitive reframing.

## Synthesis of mixed-methods findings

The qualitative findings expand upon the SEM results by providing a critical nuanced account of the statistical pathways. While the SEM demonstrates a linear relationship between academic grit and resilience (*β* = 0.436), the qualitative data reveals a “vulnerability–strength dialectic.” This suggests that the statistical path is not a frictionless transition but a high-cost process requiring students to actively manage self-doubt to maintain grit. This nuance explains why grit accounts for only a portion of the variance in resilience; some students possess the perseverance measured by the scale yet lack the cognitive reframing necessary to convert that effort into stable resilience.

Furthermore, the synthesis identifies a “strategic agency” within social connectedness that the quantitative model simplifies.

While the SEM indicates that social resources foster optimism (*β* = 0.338), the diaries show this effect is contingent on “adaptive social navigation.” The path to optimism is primarily activated when students selectively filter their networks for emotional versus instrumental support. This meta-inference suggests that the link between social connectedness and optimism is mediated by a student’s capacity for social discernment—a cognitive skill not captured by Likert-scale items. By integrating these perspectives, the study moves from a static model of “traits” to a dynamic model of “negotiation,” where student thriving is a continuous balance between external resources and internal meaning-making. Ultimately, the qualitative findings satisfy the goals of an explanatory sequential design by clarifying the mechanisms behind the statistical model.

## Discussion and conclusion

This study investigates the interplay between academic grit, social connectedness, resilience, and optimism among university students. By integrating quantitative and qualitative data, the research clarifies the dynamic relationships among these constructs and contributes to the literature on student motivation and well-being. These findings are contextually situated within the Chinese higher education landscape, where cultural and institutional norms deeply emphasize academic persistence and social interdependence.

The results notably revealed that academic grit showed a significant positive association with both resilience and optimism. This confirms that grit, operationalized here as proactive stamina and long-term passion, is linked to the psychological energy required to activate recovery processes. In the Chinese context, this grit may be reinforced by Confucian values regarding effort (*qinfen*), which frame diligence as a moral obligation and a vehicle for social mobility. Consequently, gritty students likely leverage this cultural emphasis on hard work to maintain commitment during high-pressure academic periods.

Building upon this, the current study demonstrates the significant role of grit in relation to resilience. The significant path coefficient (*β* = 0.436, *p* < 0.001) is particularly noteworthy. To address the conceptual overlap between these constructs, we interpret grit as the “behavioral engine” associated with a student to remain in the academic marathon, while resilience is the “corrective mechanism” used to navigate the hurdles within that race. Specifically, grit provides the sustained energy to remain engaged with a long-term goal (the proactive component), whereas resilience represents the reactive capacity to adapt and emotionally recover after that goal has been momentarily thwarted (the elasticity component). Recent person-centered research has further clarified this construct by identifying distinct profiles of grit, such as those characterized by both great effort and strong interest, which represent an optimal configuration of personal resources. From the perspective of Conservation of Resources (COR) theory, these high-grit profiles allow students to better manage academic demands and emotional stressors (Liu et al., 2025).

Qualitative data enrich this understanding by illustrating how persistence through academic hurdles fosters personal strength. For instance, Participant 33 (engineering) noted that completing difficult tasks felt empowering, demonstrating how grit reinforces resilience building. Statistically, grit was also positively associated with optimism (*β* = 0.276, *p* < 0.05). While previous research highlights that optimistic students often exhibit greater persistence (Bhamra et al., 2011; Tetzner and Becker, 2018), our findings suggest a bidirectional relationship where grit actively contributes to an optimistic perspective (Ruthig et al., 2007). Crucially, the significant indirect association between academic grit and optimism via resilience (*β* = 0.223, *p* < 0.001) underscores resilience as a vital intervening variable. As students persistently address challenges, they develop an adaptive capacity that fosters confidence in achieving favorable future outcomes. This dynamic was consistently reflected in student diaries, where perseverance during demanding periods helped maintain a positive outlook. Consequently, grit facilitates a long-term perspective in which academic challenges are viewed as surmountable, directly supporting an optimistic disposition (Duckworth and Quinn, 2009; Locke and Latham, 1990).

Regarding social connectedness, the robust positive relationship with resilience (*β* = 0.492, *p* < 0.001) underscores the vital role of social support in students’ academic journeys (Bailey et al., 2018; Van Bel et al., 2009). This aligns with research by Allen et al. (2008) and Meng et al. (2018), which suggests that strong social networks enable students to better navigate academic and emotional difficulties. This association is particularly salient in the collectivist Chinese context, where social interdependence and the maintenance of harmonious relationships (*guanxi*) provide a “collective safety net.” Such shared responsibility buffers individuals against the psychological toll of intense academic competition. Empirically, these findings suggest that a sense of belonging and emotional connection within social networks equips students to recover effectively from setbacks (Çiçek, 2021; Nitschke et al., 2021). Beyond peer networks, teacher support serves as a vital resource that mitigates burnout and negative emotions by fostering academic buoyancy (Derakhshan and Fathi, 2025; Li et al., 2025).

Qualitative data enrich these findings by highlighting how social networks provide both emotional and practical relief. For instance, Participant 9 (psychology) described how sharing concerns with peers mitigated stress, illustrating the buffering effect of social ties (Oddone Paolucci et al., 2021). Notably, the qualitative results revealed that students act as agents in “adaptive social navigation,” strategically seeking diverse forms of support tailored to specific challenges (Bradley et al., 2021; Oddone Paolucci et al., 2021). This flexibility emphasizes how students skillfully leverage relationships to bolster resilience (Meng et al., 2018). Quantitatively, social connectedness also showed a significant direct association with optimism (*β* = 0.338, *p* < 0.001), consistent with Phan (2016). Mirroring the results for grit, this relationship was mediated by resilience. The indirect path to optimism via resilience (*β* = 0.252, *p* < 0.001) reinforces the latter’s role as a key intervening variable; emotional resources from social bonds enhance the adaptive capacity required to maintain a positive outlook (Ergün-Başak and Can, 2018; Jose et al., 2012; Olasupo et al., 2021; Rehman et al., 2023). Qualitative entries corroborated this, as students frequently credited their social circles with helping them sustain optimism despite persistent academic pressures.

The mediation analysis identifies resilience as a central intervening variable between academic grit, social connectedness, and optimism. While these results reflect statistical associations at a single time point, they represent a logical mapping of psychological resources rather than a confirmed temporal sequence (Masten and Cicchetti, 2016). Qualitative insights extend this interpretation by framing these statistical paths as a lived process of growth. Student diaries reveal that resilience acts as a reflective space for evaluating coping strategies and converting social resources and gritty behaviors into an optimistic belief system (Datu, 2021). Within the Chinese context, these effects appear driven by culturally situated efforts to maintain psychological balance, positioning resilience as a buffer for post-adversity growth (Masten and Cicchetti, 2016; Oriol et al., 2020; Wright et al., 2012).

Framing resilience as an intervening variable treats it as a self-reflective clearinghouse where grit and support are processed, rather than a simple causal trigger (Datu, 2021). Consequently, gritty and socially connected students are better positioned to recover from setbacks, which is associated with a more optimistic mindset (Genç and Arslan, 2021; Gómez Molinero et al., 2018; Hojat et al., 2015). Furthermore, related research on academic buoyancy suggests that the intervening role of resilience identified here may sequentially reduce negative affect to facilitate optimism (Derakhshan and Fathi, 2025; Li et al., 2025). Diaries further illustrate resilience as a dynamic process developed through introspection (Luthar et al., 2000; Smith et al., 2008), exemplified by Participant 7’s shift from failure-dwelling to proactive problem-solving. This process-oriented view remains consistent with contemporary research framing resilience as a context-dependent, evolving capacity (Aburn et al., 2016; Herrman et al., 2011; Luthar et al., 2000).

In conclusion, this study explored the complex relationships between academic grit, social connectedness, resilience, and optimism among university students. Findings indicate that academic grit and social connectedness are significant predictors of resilience, which in turn intervenes in their associations with optimism. These results serve as context-situated contributions, highlighting how students within the rigorous Chinese higher education system leverage cultural norms of perseverance and collectivist social bonds to maintain an optimistic outlook.

## Implications and limitations

This research expands the scholarship on grit, social connectedness, and resilience by providing an integrated perspective on their influence on student well-being. By refining the conceptualization of resilience as both a mediator and a skill fostered through reflection, this study offers a more holistic view of academic coping than previous works that often analyzed these traits in isolation (Duckworth et al., 2007; Luthar et al., 2000). Our findings align with established frameworks, including goal-setting theory (Locke and Latham, 1990) and self-psychology (Kohut, 1984), demonstrating how perseverance and social bonds synergistically shape outcomes. Furthermore, the mixed-methods design bridges quantitative trends with qualitative depth, illustrating resilience as a dynamic, flexible capacity driven by personal experience and introspection (Smith et al., 2008). This suggests that resilience-building efforts must address both the cognitive and emotional dimensions of student development.

Practically, these results offer clear guidance for institutional policy. The significance of grit and social connectedness implies that universities should prioritize initiatives such as academic mentorship, goal-setting workshops, and peer-support networks to improve performance and well-being. Given the central mediating role of resilience, targeted interventions—including mindfulness-based cognitive therapy (MBCT), guided journaling, and cognitive restructuring—could equip students with the tools to reframe setbacks effectively. Moreover, creating inclusive campus environments that foster a sense of belonging is essential, particularly for at-risk populations like international or underrepresented students, for whom social connectedness is a critical factor in academic and social adaptation.

While this study offers valuable insights into grit, social connectedness, resilience, and optimism, some limitations should be noted. First, the quantitative analysis utilized a cross-sectional design, which identifies concurrent associations and precludes definitive causal inferences. While we specified a directional grit → resilience → optimism pathway based on the Conservation of Resources (COR) theory—which posits that foundational personal and social assets typically precede adaptive functional capacities—the current data cannot rule out reciprocal or reverse relationships. For instance, higher optimism might proactively foster greater grit and social engagement. Future longitudinal validation is required to confirm the stability and temporal order of these relationships. Because grit and resilience share overlapping characteristics—such as persistence through failure—measuring them simultaneously makes it difficult to definitively untangle their independent effects. It is possible that resilience and grit share a reciprocal relationship where adaptive recovery (resilience) further reinforces a student’s long-term passion and perseverance (grit). Although SEM suggests resilience mediates grit and social connectedness’s effects on optimism, longitudinal studies are needed to confirm the direction and stability of these relationships over time. Longitudinal designs could track changes in grit, resilience, and optimism throughout students’ academic journeys, providing deeper understanding of their evolution and impact on long-term academic outcomes.

Second, the reliance on self-report measures introduces potential social desirability bias and may oversimplify complex student experiences. Future research should incorporate a multi-method approach, integrating objective academic performance metrics and third-party assessments of social connectedness and resilience. Furthermore, experimental designs that manipulate social connectedness or implement resilience-building interventions could more rigorously test causal relationships between these variables. Third, the reliance on convenience sampling at two elite-tier universities in eastern China restricts the external validity of the findings. Since participants primarily originated from urban, middle-class backgrounds and selective high schools, they represent a “high-resource” cohort. In these contexts, social connectedness mechanisms may be more accessible or culturally emphasized than in rural or lower-tier institutional settings. Consequently, the identified pathways—especially the role of social resources in fostering resilience—might be specific to high-achievement academic cultures. Future research should utilize stratified or random sampling across a broader range of vocational and provincial colleges to determine if these psychological mechanisms persist for students with fewer socioeconomic or institutional supports.

Finally, although qualitative diary data provided rich personal insights, diary entries may have limited participants’ full expression of thoughts and emotions. Future qualitative research could employ more interactive methods, such as in-depth interviews or focus groups, to explore student experiences with resilience, social connectedness, and optimism more deeply. These methods could also allow for deeper investigation into specific strategies students use to navigate academic challenges and maintain a positive outlook.

## Data Availability

The data analyzed in this study is subject to the following licenses/restrictions: the data supporting this study’s findings are available from the corresponding author upon reasonable request. Requests to access these datasets should be directed to Li Wang, qlyyhl123@sina.com.
